# Tannic Acid-Stabilized Silver Nanoparticles Used in Biomedical Application as an Effective Antimelioidosis and Prolonged Efflux Pump Inhibitor against Melioidosis Causative Pathogen

**DOI:** 10.3390/molecules26041004

**Published:** 2021-02-14

**Authors:** Oranee Srichaiyapol, Saengrawee Thammawithan, Pawinee Siritongsuk, Sawinee Nasompag, Sakda Daduang, Sompong Klaynongsruang, Sirinan Kulchat, Rina Patramanon

**Affiliations:** 1 Department of Biochemistry, Faculty of Science, Khon Kaen University, Khon Kaen 40002, Thailand; oranee_sr@kkumail.com (O.S.); th_saengrawee@kkumail.com (S.T.); pawinee.siri@kkumail.com (P.S.); somkly@kku.ac.th (S.K.); 2 Research Instrument Center, Khon Kaen University, Khon Kaen 40002, Thailand; sawina@kku.ac.th; 3 Interdisciplinary Graduate Program in Genetic Engineering, Graduate School, Kasetsart University, Bangkok 10900, Thailand; 4 Protein and Proteomics Research Center for Commercial and Industrial Purposes (ProCCI), Khon Kaen University, Khon Kaen 40002, Thailand; sakdad@kku.ac.th; 5 Faculty of Pharmaceutical Sciences, Khon Kaen University, Khon Kaen 40002, Thailand; 6 Department of Chemistry, Faculty of Science, Khon Kaen University, Khon Kaen 40002, Thailand; sirikul@kku.ac.th

**Keywords:** melioidosis, silver nanoparticles, biofilm inhibition, mechanism, resistance induction, efflux pump inhibition, biomedical application

## Abstract

*Burkholderia pseudomallei* is the causative pathogen of melioidosis and this bacterium is resistant to several antibiotics. Silver nanoparticles (AgNPs) are an interesting agent to develop to solve this bacterial resistance. Here, we characterize and assess the antimelioidosis activity of AgNPs against these pathogenic bacteria. AgNPs were characterized and displayed a maximum absorption band at 420 nm with a spherical shape, being well-monodispersed and having high stability in solution. The average size of AgNPs is 7.99 ± 1.46 nm. The antibacterial efficacy of AgNPs was evaluated by broth microdilution. The bactericidal effect of AgNPs was further assessed by time-kill kinetics assay. Moreover, the effect of AgNPs on the inhibition of the established biofilm was investigated by the crystal violet method. In parallel, a study of the resistance induction development of *B. pseudomallei* towards AgNPs with efflux pump inhibiting effect was performed. We first found that AgNPs had strong antibacterial activity against both susceptible and ceftazidime-resistant (CAZ-resistant) strains, as well as being efficiently active against *B. pseudomallei* CAZ-resistant strains with a fast-killing mode via a bactericidal effect within 30 min. These AgNPs did not only kill planktonic bacteria in broth conditions, but also in established biofilm. Our findings first documented that the resistance development was not induced in *B. pseudomallei* toward AgNPs in the 30th passage. We found that AgNPs still showed an effective efflux pump inhibiting effect against these bacteria after prolonged exposure to AgNPs at sublethal concentrations. Thus, AgNPs have valuable properties for being a potent antimicrobial agent to solve the antibiotic resistance problem in pathogens.

## 1. Introduction

*Burkholderia pseudomallei* is the causative pathogen of melioidosis and this bacterium is resistant to several antibiotics, leading to the need for alternative agents for the treatment of melioidosis [1]. *B. pseudomallei* has recently developed resistance to several groups of antibiotics, including aminoglycosides, macrolides, quinolones, penicillin, rifamycin, as well as first, second, and third generations of cephalosporins [2]. To overcome this problem, many studies have demonstrated the better antibacterial effects of alternative agents. Antimicrobial peptides (AMPs) from human cathelicidin-derived peptides and by synthesis are one interesting group of agents. Thus, LL-37 and its variant LL-31 have been developed and have exhibited killing activity against *B. pseudomallei* [3]. Nevertheless, the researchers found that these AMPs were still sensitive to enzymatic degradation by protease in serum, causing less killing efficacy [4]. Nowadays, melioidosis patients are typically treated with ceftazidime (CAZ) [5]. The patients routinely take a high dose of CAZ for treatment. After multiple treatments, many studies have reported that *B. pseudomallei* becomes resistant to CAZ [6]. This occurrence decreases the efficacy of CAZ treatment and increases relapses in melioidosis patients. These results have led to a search for alternative agents for the treatment of melioidosis. 

One of the interesting alternative agents is metal nanoparticles, especially silver nanoparticles, on which there have been many studies [7]. The biocidal activity of silver nanoparticles against a broad spectrum of microbes has been proven [8]. Silver nanoparticles (AgNPs) are clusters of the metal silver in the range of 1–100 nm in size [9]. These kinds of NPs can be optionally synthesized by physical, chemical and biological methods. The different methods result in various shapes and sizes of NPs due to different synthesis methods, such as spherical, anisotropic, triangular nanoprisms, nanocubes and rod-shaped. Additionally, differences in particle size and shape also result in meaningfully different antibacterial activities and mechanisms of action against bacterial cells [10,11,12]. In 2016, Siritongsuk and coworkers studied the mechanism of action of AgNPs against melioidosis pathogenic cells [13]. As a result of this study, they found a two-phase mechanism of action; the first phase showed by cell death induction within the first 5-30 min by cell membrane disruption. The second phase is the ROS induction within 1-4 h, slowly disrupting the macromolecules within the bacterial cells, by which cell death is separable from the ROS induction, AgNPs mainly contributes to the killing action.

Furthermore, the efficiency of AgNPs in inhibiting the production of biofilm has been widely evaluated. Biofilm is an extracellular polymeric matrix created by bacterial cells as a community of complex bacterial populations. It creates architectural biofilms that escape from elimination by antibiotics, as well as evading the human immune system. These biofilm architectures have become a major problem, causing pathogens to be resistant to many antibiotics [14]. In a previous study, AgNPs have been approved to reduce the biofilm biomass within 24 hours through their smaller size and penetrating ability inside the established biofilm [15]. AgNPs in the sizes of 1 to 100 nanometers can inhibit the biofilm production of *P. aeruginosa* and *S. epidermidis* [16], as well as which AgNPs with an average diameter of 25.2 ± 4 nanometers can effectively inhibit the production of biofilms in *P. aeruginosa* [17]. 

Tannic acid is used as a capping agent and stabilizer in the synthesis of AgNPs providing a biocompatible nanocomposite for better antibacterial activity in various applications [18,19]. Tannic acids or tannins are phenolic compounds that are widely found in plants. These kinds of phytochemicals show a beneficial effect with rich antioxidant and antimicrobial activities. Tannic acid stabilized-silver nanoparticles present effective antimicrobial activity against various bacteria, including antibiotic-resistant strains. Furthermore, phytochemical tannic acid has been evaluated from previous work to act as an effective efflux pump inhibitor (EPI) in preventing bacterial resistance induction in prolonged exposure to a sublethal dose of the antibacterial agent [19,20]. 

Interestingly, the development of AgNPs as a novel antibacterial agent seems to be cost-effective and attractive to solve the antibiotic-resistant pathogens problem. Therefore, the present study aims to evaluate antibacterial activity along with biofilm inhibition of AgNPs against *B. pseudomallei*. In parallel, the investigation of resistance induction of this bacteria after prolonged exposure to sublethal concentrations of AgNPs, as well as prolonged activity of efflux pump inhibiting efficacy were performed.

## 2. Results

### 2.1. Physicochemical Characterization of Silver Nanoparticles 

#### 2.1.1. UV-visible Spectroscopy and Transmission Electron Microscopy

**Figure 1:**
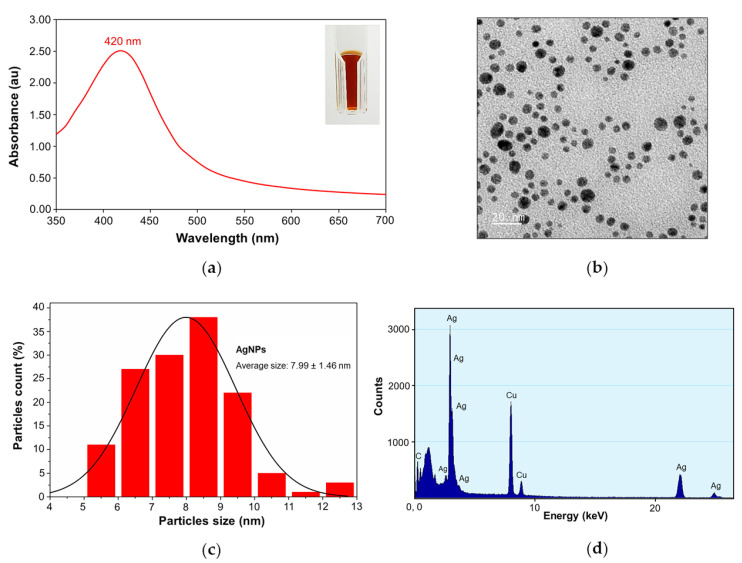
Physicochemical characterization of silver nanoparticles: (**a**) UV-Vis spectra of silver nanoparticles at 420 nm, inset: the dark yellow color of AgNPs; (**b**) transmission electron micrograph (TEM) of AgNPs showing the NPs spherical shape; (**c**) the average size of 7.99 ± 1.46 nm (*n* = 137); (**d**) and the EDX spectrum of AgNPs.

Tannic acid stabilized AgNPs (Tannin was used as both natural reducing agent and stabilizer) were provided by Prime Nanotechnology Co, Ltd. (Bangkok, Thailand). The concentration of AgNPs at 128 µg/mL was selected as the optimal concentration throughout characterization studies. The color of AgNPs colloids was dark yellow as shown in Figure 1a (inset). The UV-Vis spectrum showed a single peak of maximum absorption at 420 nm, corresponding to the surface plasmon resonance (SPR band) of AgNPs at room temperature (23.9 °C) (Figure 1a) [21]. A TEM micrograph exhibited that AgNPs were well monodispersed, as well as having a spherical shape (Figure 1b) with an average size of 7.99 ± 1.46 nm (*n* = 137) (Figure 1c). The energy dispersive X-ray (EDX) spectrum also confirmed the Ag metal composition of AgNPs as shown in Figure 1d, which has the characteristic peak of AgNPs at around 3 keV [22]. The peak of copper (Cu) was observed in the EDX spectrum due to elemental copper (Cu) that was attributed to the carbon-supported copper grid used for sample preparation.

#### 2.1.2. Dynamic Light Scattering and Zeta Potential Measurements

**Figure 2:**
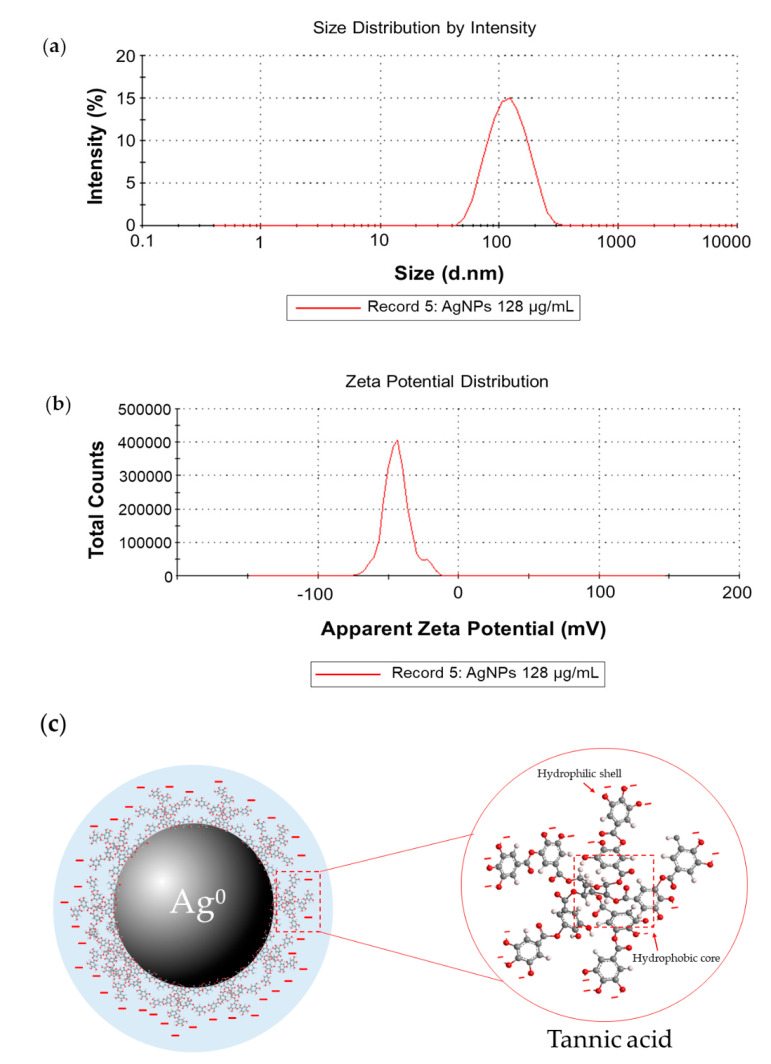
Dynamic Light Scattering (DLS) measurement of silver nanoparticles (AgNPs): (**a**) size distribution graph of AgNPs at 101.13 ± 0.85 nm; (**b**) and zeta potential of AgNPs at −47.63 ± 5.79 mV; (**c**) the simple structural model of tannic acid stabilized AgNPs molecules (pH = 5.94).

The AgNPs colloidal solutions with pH value of 5.94 were measured. Silver nanoparticles (AgNPs) had average hydrodynamic diameters of 101.13 ± 0.85 nm with low polydispersity index (PDI) value (0.20 ± 0.01 nm), indicating high particle homogeneity [23] (Figure 2a) and the zeta potential value of -47.63 ± 5.79 mV revealed the stability and negatively charge of AgNPs [24] (Figure 2b). This zeta potential value indicated that tannic acid contributed to the negative charge distribution. This distribution produced electrostatically stabilized AgNP surfaces as shown in Figure 2c, which illustrates the simple structural model of AgNPs stabilized with tannic acid [25].

### 2.2. Efficient Antibacterial Activity of AgNPs against Both Susceptible and CAZ-Resistant Strains of B. pseudomallei

**Figure 3:**
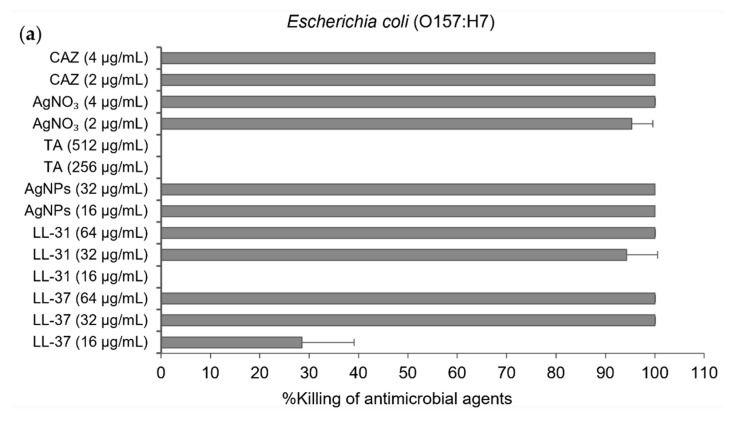

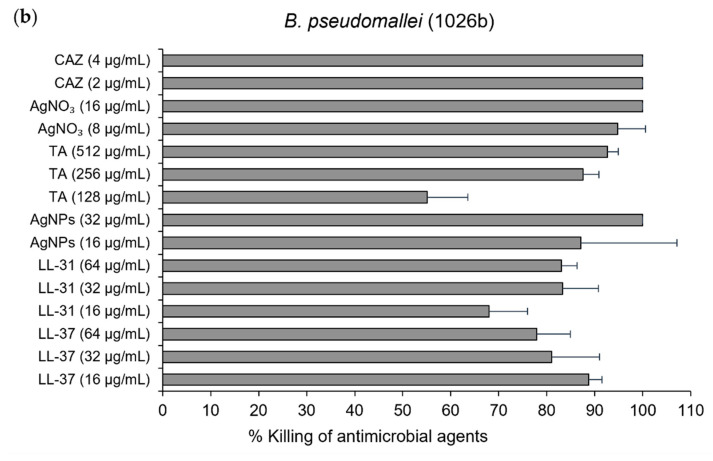
% killing of antibacterial agents, LL-37, LL-31, and AgNPs. CAZ, AgNO₃ and tannic acid (TA) were used as the positive control): (**a**) against *Escherichia coli* (O157: H7); (**b**) and *B. pseudomallei* (1026b). Data are represented as mean and standard deviation of two independent experiments performed in triplicate (*n* = 6). % killing was calculated from [1−(CFU_sample_/CFU_control_)] × 100%.

For minimum inhibitory concentration (MIC) and minimum bactericidal concentration (MBC) of antibacterial agents against *E. coli* (O157:H7) and *B. pseudomallei* (1026b), the broth microdilution method was performed. Colonies were then counted by colony plate counting assay after 20 h and incubation at 37 °C. The results showed that antimicrobial peptides LL-37could inhibit the growth of *E. coli* at the same MIC and MBC value of 32 µg/mL, whilst the MIC and MBC of LL-31 were 32 and 64 µg/mL, respectively. AgNPs also showed the same MIC and MBC value of 16 µg/mL (Table 1 and Figure 3a). For *B. pseudomallei* (1026b), AgNPs at the same MIC and MBC value of 32 µg/mL showed better bactericidal activity than both LL-37 and LL-31 (>64 µg/mL) as shown in Table 1 and Figure 3b. For the antibacterial activity test in different strains of *B. pseudomallei*, broth microdilution and resazurin colorimetric assay were performed. The MIC and MBC of AgNPs against *B. pseudomallei* strains 1026b, H777 and 316c are in the range of 32–128 µg/mL as shown in Table 2. Ceftazidime (CAZ), silver nitrate (AgNO_3_) and Tannic acid (TA) were used as the positive control. 

### 2.3. AgNPs Exhibited an Efficient, Fast Action of Bactericidal Effect Against B. pseudomallei

**Figure 4:**
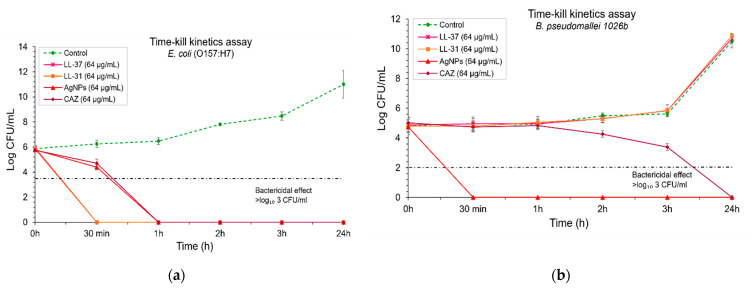
Time-kill kinetics assay of antibacterial agents (LL-37, LL-31, AgNPs and ceftazidime used as a positive control): (**a**) against *E. coli* O157: H7; (**b**) and *B. pseudomallei* 1026b. Bacterial suspensions were incubated with each antibacterial agent at a final concentration of 64 µg/ml at time points of 0, 0.5, 1, 2, 3, and 24 h. The bactericidal effect was defined as ≥ 3 log₁₀ CFU/mL compared with untreated control. Data represent mean value ± SD (error bar) from two independent experiments carried out in triplicate (*n* = 6).

To determine the bactericidal activity of antibacterial agents over time, a time-kill kinetics assay was performed. Bacterial suspensions were incubated with each antibacterial agent at the final concentration of 64 µg/mL at the time points of 0, 0.5, 1, 2, 3, and 24 h. The results show that LL-37 and LL-31 exhibited a rapid bactericidal effect against *E. coli* within 30 min, whereas AgNPs and ceftazidime (as a positive control) could kill *E. coli* within 1 h (Figure 4a). To determine these time-kill kinetics assay against *B. pseudomallei*, the results showed that AgNPs could kill *B. pseudomallei* strongly within 30 min, whereas ceftazidime could slowly kill this pathogenic bacterium within 24 h. On the other hand, both LL-37 and LL-31 at a concentration of 64 µg/mL could not inhibit *B. pseudomallei* at all (Figure 4b).

**Figure 5:**
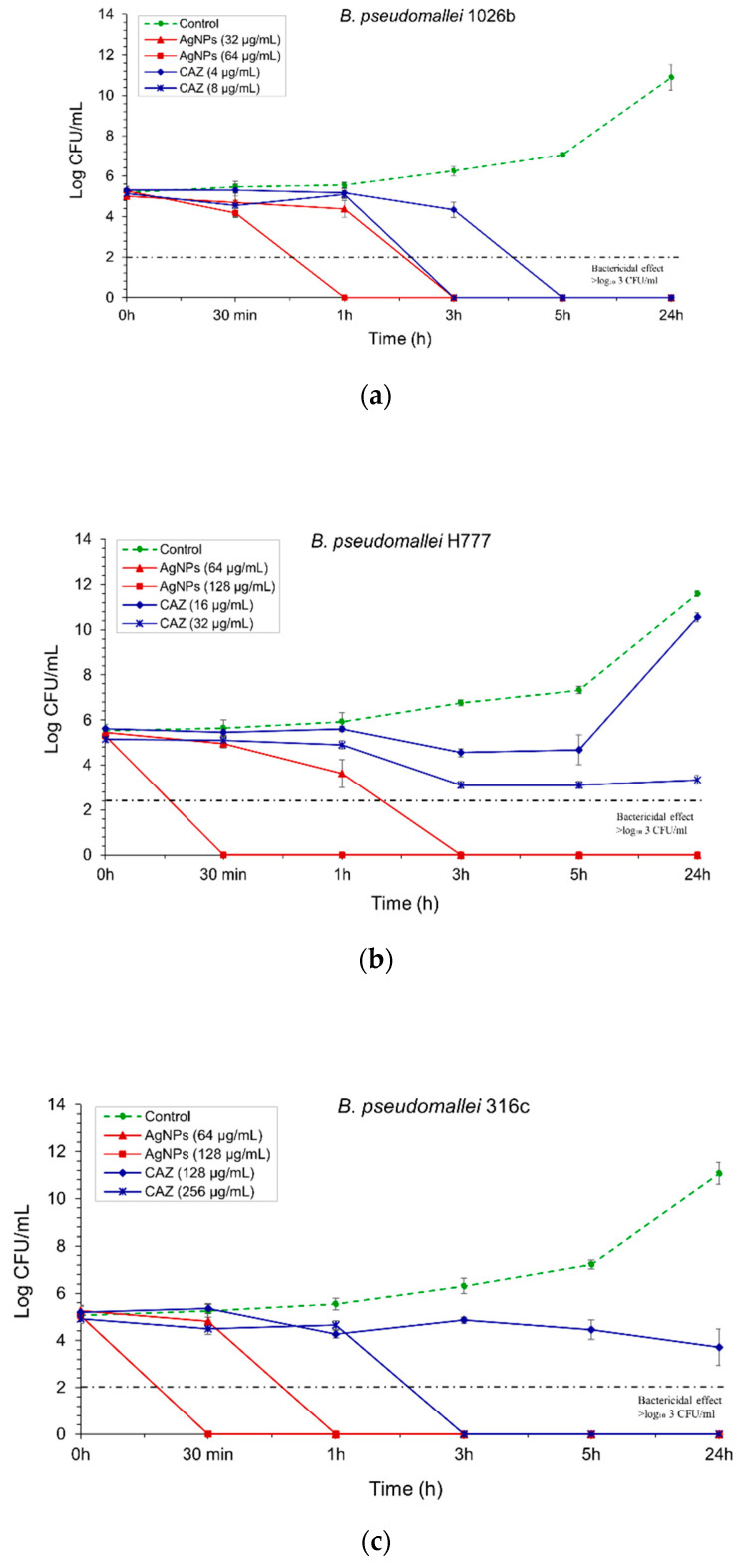
Time-kill kinetics assay of AgNPs and CAZ: (**a**) against *B. pseudomallei* (1026b); (**b**) H777; (**c**) and 316c. The bacterial suspension was performed at the time points of 0, 0.5, 1, 3, 5 and 24 h. The bactericidal effect was defined as ≥3 log₁₀ reductions in the colony-forming unit (CFU/mL), compared with untreated control. Data are represented as mean value ± SD (error bar) from two independent experiments carried out in triplicate (*n* = 6).

The bactericidal effect of AgNPs was particularly determined against three isolates of *B. pseudomallei*: 1026b represented the CAZ-nonresistant isolate, H777 is moderately CAZ-resistant and 316c is the highly CAZ-resistant isolate. For the 1026b isolate, 32 µg/mL of AgNPs could exhibit bactericidal effect as effectively as ceftazidime (CAZ) within 3h, whilst AgNPs at 64 µg/mL showed complete bactericidal effect within 1 h (Figure 5a). For the H777 isolate, AgNPs at 64 µg/mL slowly inhibited this pathogenic isolate within 3 h, whilst the bactericidal effect could be seen within 30 min when the concentration of AgNPs was increased to 128 µg/mL (Figure 5b). For 316c isolate, AgNPs at 128 µg/mL could exhibit a strong bactericidal effect within 30 min, whilst CAZ at the same concentration with AgNPs could not completely inhibit this isolate within 24 h (Figure 5c).

### 2.4. AgNPs Can Inhibit Biofilm Formation of B. pseudomallei

**Figure 6:**
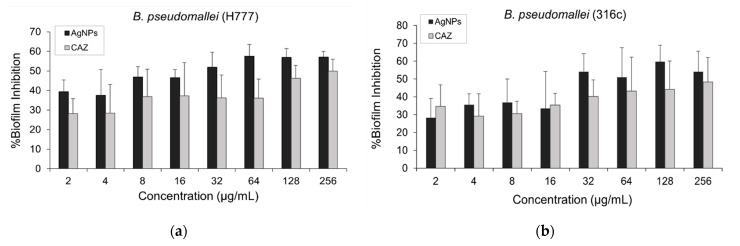
The effect of AgNPs and CAZ on inhibition of established biofilm against both CAZ-resistant strains: (**a**) *B. pseudomallei* (H777); (**b**) and 316c. Data are represented in mean ± SD from at least two independent experiments (*n* = 5).

The effect of AgNPs on the inhibition of established biofilm against CAZ-resistant strains of *B. pseudomallei* (H777 and 316c) was performed by crystal violet assay. From the results as shown in Figure 6a,b, AgNPs showed an increasing trend of % biofilm inhibition against both strains when the concentration was increased. We found that at least 32 µg/mL of AgNPs exhibited biofilm inhibition >50% against both CAZ-resistant strains.

### 2.5. No Resistance Induction Developed by B. pseudomallei towards AgNPs after 30th Bacterial Generation Passage

To study the resistance induction trend of *B. pseudomallei*, the nonresistant (1026b), moderately CAZ-resistant (H777) and highly CAZ-resistant strains (316c) were tested after prolonged exposure to sub-MIC concentrations over time with AgNPs. The MIC values before passage and after the 30th passage are shown in Table 3. All three different strains of *B. pseudomallei* had no resistance development against AgNPs, as well as those of CAZ when the MIC values before and after the 30th passage were reported as the same value. The culture purity of all strains on ASH media in every 10th generation passage is provided in Figure S1.

### 2.6. AgNPs Still Showed Prolonged Efflux Pump Inhibiting Effect against B. pseudomallei via Phenotypic EtBr-agar Cartwheel Assay after 30th Bacterial Generation Passage

**Figure 7:**
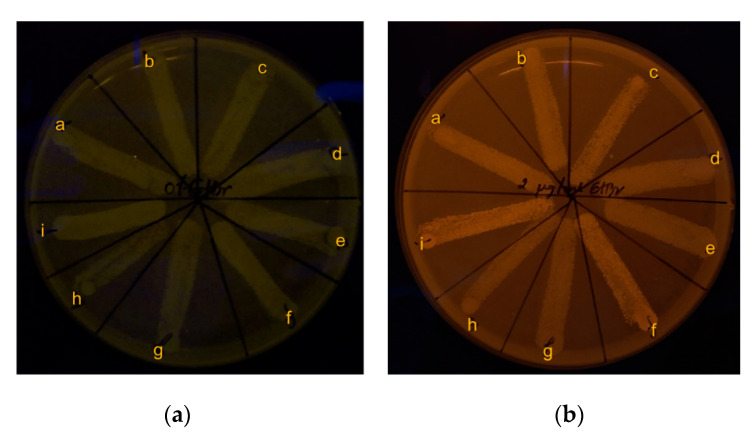
Phenotypic efflux pump activity of *B. pseudomallei* by EtBr-agar cartwheel assay: (**a**) 0% EtBr agar (control); (**b**) 2 µg/mL EtBr agar. Broth conditions for 30th generation passage: image overlay: a) *B. pseudomallei* 1026b in untreated control broth; b) broth supplemented with ¼ MIC of CAZ; c) broth supplemented with ¼ MIC of AgNPs. For H777: d) untreated control broth; e) broth supplemented with ¼ MIC of CAZ; f) and broth supplemented with ¼ MIC of AgNPs. For 316c: g) untreated control broth; h) broth supplemented with ¼ MIC of CAZ; i) and broth supplemented with ¼ MIC of AgNPs.

To detect the efflux pump activity, cartwheel assay was carried out. Zero % of Ethidium bromide (EtBr) agar was used as control (Figure 7a). The results showed no fluorescence emission of bacteria on 2 µg/mL EtBr agar. This result indicates that three different strains of *B. pseudomallei* before and after the 30th generation passage had efflux pump activity to pump EtBr (as an efflux pump substrate) out of the cells [26] (Figure 7b). 

**Figure 8:**
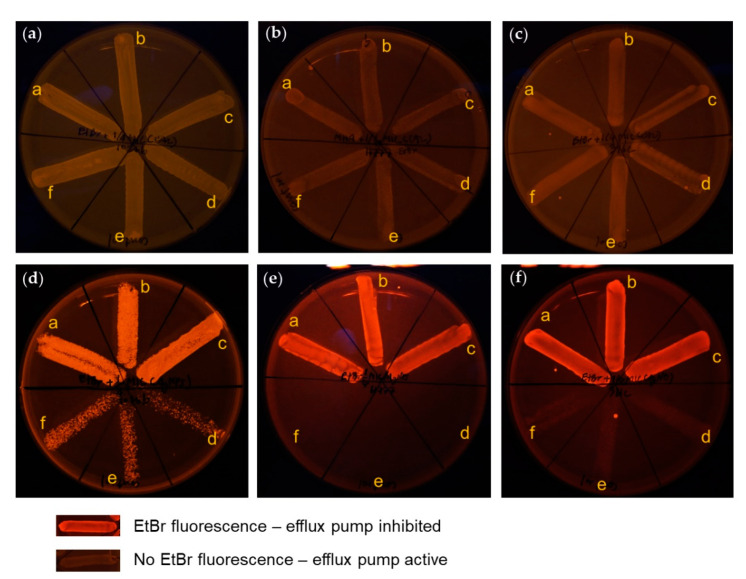
Phenotypic efflux pump inhibiting effect of antibacterial agents: (**a–c**) 2 µg/mL EtBr-agar supplemented with ¼ MIC of CAZ; (**d–f**) 2 µg/ml EtBr-agar supplemented with ¼ MIC of AgNPs; (**a**,**d**) against *B. pseudomallei* strain 1026b; (**b**,**e**) H777; (**c**,**f**) and 316c. Broth conditions for 30th bacterial generations passage: (inset, **a**–**c**) *B. pseudomallei* in broth supplemented with ¼ MIC of each agent; (inset, **d**–**f**) and *B. pseudomallei* in untreated control broth.

To investigate the efflux pump inhibiting effect of AgNPs, a cartwheel assay was performed as mentioned above. The results show that ¼ MIC concentration of AgNPs supplemented in EtBr-agar media highly inhibited the efflux pump in three different strains of *B. pseudomallei*, both before and after the 30th passage (Figure 8d–f), whilst all tested bacteria treated with ¼ MIC concentration of CAZ supplemented in EtBr-agar media had no fluorescent emission, indicating the efflux pump was active (Figure 8a–c). These fluorescence emission phenomena by EtBr in all strains treated with AgNPs indicated that AgNPs might be an effective efflux pump inhibitor. AgNPs might target the inhibiting transmembrane protein responsible for efflux pump activity, causing EtBr accumulation within the bacterial cell. Thus, fluorescent emission can be seen under UV light [27]. 

### 2.7. AgNPs Acted as an Effective Efflux Pump Inhibitor by Exhibiting a Maximum of 16-Fold Reduction in Efflux Pump Substrate MIC (EtBr)

To evaluate the efflux pump inhibiting effect of AgNPs quantitatively, the MIC value of EtBr with and without AgNPs before and after the 30th generation passage was found. The MIC results revealed that the MIC value of EtBr was reduced by 16-fold, 8-fold and 4-fold in the presence of AgNPs when compared with EtBr alone against *B. pseudomallei* strains 1026b, H777 and 316c, respectively. The MIC value of EtBr was reduced only 4-fold in the presence of CAZ, when compared with EtBr alone in all strains (Table 3). These results indicate that AgNPs are efflux pump inhibitors exhibiting a maximum 16-fold reduction in efflux pump substrate (EtBr) MIC. The inhibition of EtBr efflux supports the hypothesis of the efflux pump inhibiting the effect of AgNPs [28].

## 3. Discussion

Several novel antimicrobial agents need to be developed for the treatment of infectious diseases caused by pathogens and the growing problem of bacterial resistance [14,29]. Currently, nanotechnology has become an interesting area and is used in several fields. In the medical field, silver nanoparticles (AgNPs) are reported extensively as the most promising antimicrobial agent to overcome drug-resistant pathogens, as well as to develop treatments of various infectious diseases [30]. Moreover, tannic acid stabilized silver nanoparticles have been proved by many researchers to have antimicrobial efficacy against both Gram-positive and Gram-negative bacteria [31] and these AgNPs were also used in this work. We received the tannic acid stabilized silver nanoparticles used in this study from our collaborative company (Prime Nanotechnology, Thailand, co. Ltd.). Since tannin is a natural compound and is biocompatible, it was used as a reducing agent and stabilizer in their one-pot synthesis. The use of tannic acid as a reducing agent in the synthesis of AgNPs provides colloidal solutions with more stability. The size of AgNPs can be controlled by molar ratio variation of tannic acid (TA) to silver nitrate (AgNO_3_). AgNPs were synthesized over a wide range of values of the initial molar ratio of TA to AgNO_3_. The more molar the ratios of TA/AgNO_3_ are increased, the greater the increase in particle size of AgNPs [32,33]. AgNPs were characterized by UV-Visible spectroscopy and showed a maximum absorption peak at 420 nm (Figure 1a) which was similar to the results reported by Sharma et al. [21]. The monodispersed spherical shape and size distribution was analyzed by TEM and found an average size of 7.99 ± 1.46 nm for AgNPs. The silver element composition in AgNPs was confirmed by EDX, revealing a major peak at around 3 keV [22]. From Dynamic Light Scattering (DLS) measurement, we found that the hydrodynamic diameter of AgNPs was greater than the average size observed from TEM due to the solvent and stabilizer layers present around the AgNPs’ surface [34]. The negative zeta potential of −43.9 mV (Figure 2b) proved the high stability of AgNPs due to the electrostatic repulsion of NPs in the solution [24,35,36]. 

The biological action of AgNPs depends on several factors including size, shape, surface charge, size distribution, particle morphology, as well as the type of reducing agents used in the synthesis of AgNPs [11,18,37,38]. The zeta potential value of AgNPs is −47.63 ± 5.79 mV, indicating the surface of AgNPs was negatively charged. This value indicated that tannic acid contributed to the negative charge. This distribution produced electrostatically stabilized on AgNPs surface (Figure 2c) [25]. The hydrophobic moieties and hydrophilic shell of tannic acid stabilized on AgNPs play an important role for its interaction with the hydrocarbon chain of lipid, as well as surface proteins on bacterial cells. These polyphenolic features of tannic acid promote close contact between AgNPs and the bacterial cell membrane [25,37,39]. As far as size and shape are concerned, particles of a smaller size with good monodispersal of AgNPs seem to be more effective and have superior properties [18]. Many previous studies have indicated that the bactericidal properties of nanoparticles are size-dependent [7,40,41,42]. The researchers have identified that AgNPs act primarily in three possible mechanisms against Gram-negative bacteria: (1) nanoparticles (mainly in the range of 1–10 nm) attach to the surface of the cell membrane, cause permeability and disrupt the respiration in bacteria; (2) AgNPs penetrate inside the bacterial cell and cause serious damage by interacting with sulfur- and phosphorus-containing macromolecules; (3) AgNPs release silver ions, which contribute to the bactericidal effect of the AgNPs [38,43]. In addition, the antibacterial activity of AgNPs is not only size, but also shape-dependent. The results from the study of Pal and coworkers [11] reported that the bactericidal properties of silver nanoparticles undergo a shape-dependent interaction with the *Escherichia coli*. The nanoscale size and the presence of a {111} plane of AgNPs combine to promote the biocidal property as well.

For antibacterial investigation of *E. coli* and *B. pseudomallei*, the difference in active concentration is due to the different modes of action on bacterial cells between antimicrobial peptide LL-37 and AgNPs. AgNPs possess lower antibacterial concentration than those of LL-37 because AgNPs act as a nonspecific multimodal mechanism of action both insight and outsight of the bacterial cells. Thus, this advantage allows their effective antibacterial action at very low concentrations [44,45]. On the other hand, LL-37 is a cationic peptide, and the biological function of this peptide has been debated in several studies [3,46,47]. Many studies have been conducted about the possible mechanism of action, such as a barrel-stave conformation, toroidal pore formation and the carpet model. However, the antibacterial role of LL-37 is assumed to act by a specific mode of action on bacterial cell membrane. The limitations of the specific mode of action probably depend on the interaction between negatively charged bacterial cell membrane and negative or positive charge of antimicrobial peptides. So, a large amount of LL-37 is needed for use to inhibit bacteria at the same bacterial concentration as the AgNPs used [46]. Moreover, we investigated the antibacterial activity of tannic acid. The results show that tannic acid itself has no bactericidal effect even at high concentration (>512 µg/ml) against all strains of pathogens, which was similar to the previous report [37]. These results indicate that the main bactericidal effect is exhibited by AgNPs. In this study, we testified the efficiency of the bactericidal activity of AgNPs against three different strains of *B. pseudomallei*, including susceptible, moderately, and highly CAZ-resistant isolates. We found that AgNPs exhibited strong antibacterial activity against all strains of *B. pseudomallei* as in the results reported previously [13]. The time-dependent bactericidal effect can be monitored by time-kill kinetics curves of bacterial growth and death to evaluate the effect of AgNPs over time [48,49]. Our time-kill kinetics results revealed that AgNPs showed a fast mode of killing action within 30 minutes, mainly with *B. pseudomallei* CAZ-resistant strains. AgNPs showed a faster bactericidal effect than those of CAZ. Moreover, several reports have demonstrated that *B. pseudomallei* can produce biofilms to survive and protect their bacterial communities from environmental fluctuations both in vitro and in vivo [50]. The biofilm of *B. pseudomallei* has been reported as one of the virulence factors which allows these bacteria to be pathogenic. They are significantly resistant to various antibacterial agents as well as active antibiotics, such as tested CAZ, imipenem and trimethoprim/sulfamethoxazole, as compared with planktonic bacterial cells in the same isolates [3,51]. In the present study, we found that at least 32 µg/ml of AgNPs showed >50% biofilm inhibition against both CAZ-resistant strains. AgNPs could reduce the increase in planktonic bacterial cell number, which could produce the biofilm. The previous evidence documented that AgNPs could hinder the inhibition activity due to AgNP agglomeration. Moreover, being negatively charged themselves, they are electrostatically repulsed from the negatively charged surface of the bacterial cell, thus AgNPs might penetrate to the extent of biofilm as much as half of all existing biofilms [52]. Nevertheless, we first found that AgNPs showed more potentially inhibited established biofilm of *B. pseudomallei* than those treated with CAZ at the same concentration. 

According to an interesting point from the biofilm inhibition results, we assume that the remaining bacteria can survive in their produced biofilm. This stage could significantly induce bacterial resistance consistent with a previous study [51]. Therefore, in parallel, we further cultured bacteria in vitro. We allowed them to grow in natural conditions with a passage in a tube of fresh broth containing sub-MIC of AgNPs and another tube of broth containing sub-MIC of CAZ for 30th generation passage testing. The screening test for investigating the resistance induction was then performed. Interestingly, we first found that *B. pseudomallei* was efflux pump active in the case of testing with CAZ but revealed an efflux pump inhibiting effect against AgNPs. Ethidium bromide (EtBr) has been reported as the substrate in many efflux pump systems in both Gram-positive and Gram-negative bacteria. Thus, it has been used to intrinsically evaluate efflux pump activity in *Escherichia coli (E. coli)*, *Staphylococcus aureus (S. aureus)*, *Klebsiella pneumoniae (K. pneumonine)*, *Pseudomonas aeruginosa (P. aeruginosa)* and *Burkholderia species* [53,54,55]. Bacteria could develop resistance by using transport proteins involved in the extrusion of antibiotics out of the cell. The expression of efflux pumps of antibiotics from the cellular milieu has been described in many reports as one of the major resistance mechanisms [56]. In the case of being efflux pump active in all strains of *B. pseudomallei* tested with CAZ, this result was similarly reported by Podnecky et al. [55] who stated that *B. pseudomallei* has an efflux pump system called the resistance nodulation cell division (RND) family which is a significant player in several drug resistances. According to the interesting results from our study, we found an efflux pump inhibiting effect of AgNPs against all strains of *B. pseudomallei*. To date, previous reports have proved that the use of metal NPs can cause the loss of proton motive force (PMF), which is essential for the normal functioning of many bacterial efflux pumps [57]. The study documented by Mishra et al. [58] found that AgNPs exhibited modulatory effects on the AcrAB-TolC efflux pump in MDR *Enterobacter cloacae*. Moreover, AgNPs also disrupted the MexAM-OPrM efflux pump kinetics in *P. aeruginosa* by terminating the proton gradient and deteriorating the PMF of the efflux pump system [59]. 

However, the phenomenon of resistance induction still did not show in the 30th generation in both CAZ and AgNPs. In this study, we focused on AgNPs, and the phenomenon of slow resistance induction might occur from the strong antibacterial activity of AgNPs together with tannic acid capping as a potential stabilizer [19]. Many studies have documented that bacteria could not develop resistance to AgNPs when compared with antibiotics, as the AgNPs can attach directly with multiple targets in the bacterial cell, causing the bacterial cell difficulty in developing resistance. This mechanism has been confirmed [60,61]. The resistance induction result from this study is consistent with a previous report on tannic acid acting as an efflux pump inhibitor (EPI), otherwise known as a resistance modulator. It is effective to use this EPI as a capping agent against resistant strains when culturing the bacteria in sub-MIC prolonged exposure to antimicrobial agents [20]. In a previous supportive study, the antibacterial agents combined with an EPI could enhance antibacterial activity and reduce the frequency of resistant induction incidence [55]. Here, we found an interesting new finding that has never been documented. Tannic acid-stabilized silver nanoparticles exhibited effective antibacterial, antibiofilm efficacy, as well as slowly inducing resistance. Furthermore, they act as a prolonged efflux pump inhibitor against CAZ nonresistant, moderately resistant, and highly resistant isolates of *B. pseudomallei* after prolonged exposure to sublethal concentrations. 

## 4. Materials and Methods

### 4.1. Bacterial Strain and Culture Media

Three strains of *B. pseudomallei* were kindly provided by Dr. Suwimol Taweechaisupapong, from the Melioidosis Research Center, Faculty of Medicine, Khon Kaen University (Khon Kaen, Thailand). The bacterial culture media used mainly in this research were Muller Hinton Broth (MHB, HiMedia Laboratories Pvt. Ltd., Bengaluru, India).

### 4.2. Antibacterial Agents and Preparations

Antimicrobial peptides (AMPs), both LL-37 and LL-31, were purchased from GL Biochem (Shanghai, China). All AMPs were prepared in sterile deionized water at 2.5 mg/ml as stock solution, then aliquoted and stored at −20 °C. One of the AMP stock aliquots was serially diluted by two-fold dilution in the range at a final concentration of 4–512 µg/mL. These AMP solution tubes were kept at −4 °C until use. Tannic acid stabilized AgNPs were given by our collaborator Prime Nanotechnology Co., Ltd. (Bangkok, Thailand) with a stock concentration of 10,000 mg/L. For AgNP solution preparation in our experiments, 1 mg/ml as a stock solution was prepared in sterile deionized water. AgNPs were then serially diluted by two-fold dilution in the range of final concentrations of 4–512 µg/mL, then kept at room temperature until used. Ceftazidime antibiotic (CAZ) was kindly provided by the Melioidosis Research Center, Faculty of Medicine, Khon Kaen University (Khon Kaen, Thailand). The preparation procedure of CAZ was the same method as the preparation method above.

### 4.3. Characterization of AgNPs

AgNPs were prepared in sterile deionized water (DI) and diluted to reach the final concentration of 128 µg/ml. The plasmon extinction spectra of AgNPs were performed by UV-Vis spectrophotometer (SpectraMax M5 Multi-Mode microplate readers, Molecular Devices, San Jose, CA, USA). The AgNPs were dropped on Formvar/carbon coated-copper grid (200 mesh) and dried overnight in a desiccator before TEM observation. Transmission electron micrographs of AgNPs were produced under the transmission electron microscope (Hitachi Model H-7650, Hitachi, Tokyo, Japan) operating at 100 kV. The dimensions of AgNPs were analyzed by Image J software, Java developed by the National Institute of Mental Health [62]. AgNPs were added in disposable zeta cells with gold electrodes at room temperature for size and zeta potential distribution analysis. Size distributions and zeta potentials of AgNPs were measured by Zetasizer Nano ZS. (Malvern, England).

### 4.4. Minimum Inhibitory Concentration (MIC) and Minimum Bactericidal Concentration (MBC) Determination by Broth Microdilution and Resazurin Colorimetric Assay 

MICs and MBCs of all antimicrobial agents against *B. pseudomallei* (strain 1026b) and *E. coli* (O157: H7, used as a comparative reference bacteria) were performed by the broth microdilution method recommended by the Clinical and Laboratory Standards Institute [63] and described by Irazazabal, et al. [64]. Briefly, bacterial cultures were adjusted in MHB to McFarland 0.5 turbidity standard (to reach 1–5 × 10^5^ CFU/mL). The equal volume of each antibacterial agent (50 µL) at a final concentration of 4, 8, 16, 32, 64, 128, 256, 512 µg/mL and bacterial solution (50 µL) were added in each well of the 96-well plate. Ceftazidime antibiotic was used as a positive control. The plate was then incubated in an incubator at 37 °C for 18–24 h. MIC and MBC determination in different strains of *B. pseudomallei* (1026b, H777 and 316c) with broth microdilution and resazurin colorimetric assay were proposed by Silveira, et al. and Teh, et al. [65,66]. Broth microdilution was carried out with the same procedure as above. After overnight incubation, 0.01% resazurin (7-hydroxy-3*H*-phenoxazine-3-one 10-oxide; Sigma-Aldrich) was added to all wells and incubated at 37 °C for another 4 h. The color change was observed. The lowest concentration before the color change was determined as the MIC, the blue color represented no growth of bacteria and the pink one meant bacterial survival. The tests were performed in at least two independent experiments in triplicates. 

### 4.5. Serial Colony Plate Counting Assay

For MIC and MBC test of various antibacterial agents against *E. coli* (O157:H7) and *B. pseudomallei* (1026b), colony counting assay was performed according to the method of Sengyee, et al. [67]. After 18–24 h incubation, the treated bacterial solution in each well was measured by serial colony plate counting assay. Briefly, 10 µL of no turbidity in the well was dropped on Muller Hinton Agar (MHA) and incubated overnight at 37 °C. The MIC was defined as the lowest concentration which could inhibit 99% of bacterial growth, whilst MBC was defined as the lowest concentration which could inhibit 100% of bacterial growth. Colonies were counted and calculated for the percentage of inhibition from:

(1) $$\mathrm{Inhibition}\;\%=\left[1-\left(\frac{{\mathrm{CFU}}_{\mathrm{test}}}{{\mathrm{CFU}}_{\mathrm{control}}}\right)\right]\;\times \;100\%$$

Each MIC and MBC was carried out in two independent experiments performed in triplicates.

### 4.6. Time-Kill Kinetics Assay

The kinetics of time-kill change of antibacterial agents against three different strains of *B. pseudomallei* and *E. coli* (O157:H7) was used to determine the potential of each antibacterial agent in killing, along with the bacterial growth curve following the procedure established by Pankey, et al. [68]. The single colony of *B. pseudomallei* and *E. coli* was cultured in Muller Hinton Broth (MHB) overnight at 37 °C. The overnight bacterial solution was then subcultured into 5 mL fresh MHB and further incubated for 1.5–2 h at 200 rpm to yield the mid-log growth phase. The 1% inoculum was adjusted into fresh MHB and 250 µL of the final concentration of 1–5 × 10^5^ CFU/ml of bacterial solution was added into equal volumes of 250 µL of each antibacterial agent. The solution with treated bacteria was incubated at 37 °C and shaken at 180 rpm in a shaking incubator. The time-kill kinetics was determined at the time points of 0, 30 min, 1, 3, 5 and 24 h. Serial 10-fold dilution was used to count the bacteria at each time point. Bactericidal activity was defined as a reduction of 99.9% or ≥ 3 log10 CFU/mL when compared with untreated control.

### 4.7. Biofilm Inhibition by Crystal Violet Assay 

Crystal violet assay is a colorimetric measurement used to stain and quantify the biofilm. Biofilm inhibition of AgNPs against *B. pseudomallei* strain H777 and 316c were performed by crystal violet assay adapted from Kunyanee, et al. [69]. Briefly, bacteria were cultured in fresh prepared Modified Vogel Bonners medium (MVBM) at 37 °C in a shaking incubator at 200 rpm. Two percent *v/v* inoculum was then transferred to fresh MVBM and incubated for another 18 h. After 18 h incubation, bacterial suspension was adjusted to reach OD_540nm_ at 0.08. Then, 200 µL of bacterial suspension was added into a 96-well plate and incubated for another 3 h at 37 °C. Nonadhering cells were removed, then replaced with fresh MVBM, and incubated further at 37 °C. After 21 h incubation, they were washed three times with sterile distilled water. The concentration of AgNPs and CAZ in the range of 2–256 µg/mL was added into a 96-well plate and incubated overnight at 37 °C. After that, they were washed three times with distilled water and attached bacteria were fixed with absolute methanol for 15 min. After being air-dried, the attached biofilms were stained with 2% *w/v* crystal violet for 15 min. Dye bound cells were then solubilized with 33% *v/v* glacial acetic acid. Each well was colorimetrically measured at 630 nm. The percentage of biofilm inhibition was calculated from:

(2) $$\mathrm{biofilm}\;\mathrm{inhibition}\%=\left[\frac{\left({\mathrm{OD}}_{\mathrm{control}}-{\mathrm{OD}}_{\mathrm{test}}\right)}{{\mathrm{OD}}_{\mathrm{control}}}\right]\;\times \;100\%$$

as proposed by Lemos, et al. [70].

### 4.8. Resistance Induction Study

To investigate resistance induction of bacteria after prolonged exposure with a sublethal concentration of AgNPs, *B. pseudomallei* strains 1026b, H777 and 316c were treated by frequent passaging in MHB, supplemented with a sublethal dose below MIC (¼ MIC of AgNPs and ¼ MIC of CAZ used as control) adapted from Elbehiry, et al. [71]. Each independent lineage was passaged in MHB supplemented with sub-MIC (¼ MIC) of AgNPs at 10-day intervals for the 30th generation passage. Culture purity was tested every 10 passages on Ashdown’s selective media specific for *B. pseudomallei*. The MIC values before passage and MIC after the 30th generation passage of AgNPs were determined by Re-MIC assay. 

### 4.9. Phenotypic Efflux Pump Activity and Efflux Pump Inhibition Detection by EtBr-Cartwheel Assay 

In the bacterial cell, ethidium bromide (EtBr, Sigma-Aldrich) has been recognized as a substrate for many efflux pump systems. The cartwheel method is a simple phenotypic test to detect the efflux pump activity of bacteria in pumping EtBr out of the cell and was used in this study according to the procedure of Anbazhagan, et al. [54]. Muller Hinton agars containing EtBr in the range of 0–2.5 µg/mL were freshly prepared on the day of the experiment. A final concentration of approximately 10^6^ CFU/mL of *B. pseudomallei* (1026b, H777 and 316c) was swabbed on the MHA-EtBr plate in a cartwheel pattern from the center to the margin of the plate. All tested plates were kept in the dark with overnight incubation at 37 °C. The plates were then visualized under UV transilluminator (Dark Reader DR46B transilluminator, Clare Chemical Research, CO, USA). No fluorescent emission was considered as being efflux pump active. For efflux pump inhibiting effect determination, an alternative agent acting as an efflux pump inhibitor (EPI) can cause EtBr accumulation within the bacterial cell and show fluorescent emission under UV light. To test the efflux pump inhibiting effect of AgNPs, a cartwheel assay was performed with the slight modification described by Christena, et al. [27]. MHA plates were supplemented with 2 µg/mL EtBr and ¼ MIC concentration of AgNPs or CAZ (used as control). *B. pseudomallei* strains 1026b, H777 and 316c were cultured in MHB containing ¼ MIC concentration of AgNPs and CAZ and incubated at 37 °C overnight. Bacterial suspension was adjusted to 0.5 McFarland standard turbidity, treated with fresh MHB containing ¼ MIC of AgNPs and CAZ for another 1 h. The treated bacteria were then swabbed in a cartwheel pattern, incubated and detected under UV light with the same procedure mentioned above. Efflux pump inhibiting effect can be detected when fluorescent emission is shown under a UV transilluminator. 

### 4.10. Efflux Pump Inhibition Evaluation and Fold Reduction in MIC by Microdilution Assay 

For quantitative evaluation of efflux pump inhibition of AgNPs, microdilution assay was assessed with the slight modification previously described by Behdad, et al. and Silverira, et al. [28,65]. AgNPs, CAZ and EtBr were prepared in the concentration range of 0.125 to 512 µg/mL. MIC determination of all agents was tested in 96-well plates and incubated overnight at 37 °C. After incubation, 0.01% resazurin was added at 10 µL in each well and incubated for another 3 h. The efflux pump inhibiting effect is determined when the MIC value of AgNPs with EtBr is lower than the MIC value of EtBr alone.

### 4.11. Statistical Analysis

All experiments were performed with at least two independent experiments in triplicate. All results are shown as mean ± standard deviations (SD) and were analyzed using Statistical Package for the Social Science (SPSS) version 16.0 (SPSS Inc., Chicago, IL, USA). 

## 5. Conclusions

AgNPs were characterized and assessed for antimelioidosis activity against melioidosis pathogenic bacteria. The characterization of AgNPs by UV-Vis spectroscopy showed the maximum absorption band at 420 nm. The average size of AgNPs was 7.99 ± 1.46 nm with a spherical shape, well-monodispersed and having high stability in solution. In our study, AgNPs possess efficient antibacterial activity and biofilm inhibition against both susceptible and CAZ-resistant strains of *B. pseudomallei*. They showed efficient activity against CAZ-resistant strains with a fast-killing mode via bactericidal effect within 30 min. Interestingly, resistance development was not induced in *B. pseudomallei* toward AgNPs. In addition, these NPs still exhibited a strong efflux pump inhibiting effect against these pathogens even after prolonged exposure for 30 passages in sublethal dose conditions. To the best of our knowledge, AgNPs have the potential to be developed as an alternative agent for melioidosis treatment due to their antimelioidosis action, slowly induced resistance, as well as effective efflux pump inhibitor properties.

## Figures and Tables

**Table 1:** Minimum inhibitory concentration (MIC) and minimum bactericidal concentration (MBC) of antibacterial agents against *E. coli* (O157:H7) and *B. pseudomallei* (1026b) after 20 h incubation at 37 °C, performed with the microdilution method and colony plate counting assay.

Antibacterial Agents (µg/mL)	*Escherichia coli* (strain O157:H7)		*Burkholderia pseudomallei* (strain 1026b)	
	MIC ^2^	MBC ^3^	MIC	MBC
LL-37	32	32	>64	>64
LL-31	32	64	>64	>64
AgNPs	16	16	32	32
AgNO_3_	4	4	16	16
Tannic acid (TA)	>512	>512	>512	>512
Ceftazidime (CAZ) ^1^	2	2	2	2

^1^ CAZ was used as a standard antibacterial agent; ^2^ MIC was defined as the lowest concentration of agent that could inhibit >99% of bacterial growth; ^3^ MBC was defined for 100% inhibition of bacteria.

**Table 2:** MIC and MBC of AgNPs and CAZ against *B. pseudomallei* strains 1026b, H777 and 316c after 20 h incubation at 37 °C performed with the microdilution method and resazurin colorimetric assay.

Antibacterial Agents (µg/mL)	*B. pseudomallei* (Strains)					
	1026b ^1^		H777 ^2^		316c ^3^	
	MIC	MBC	MIC	MBC	MIC	MBC
AgNPs	32	32	64	128	64	128
AgNO₃	16	16	16	16	16	16
Tannic acid (TA)	>512	>512	>512	>512	>512	>512
Ceftazidime (CAZ)	2	2	4	4	64	128

^1^ CAZ nonresistant isolate; ^2^ CAZ moderately resistant isolate; ^3^ CAZ highly resistant isolate.

**Table 3:** Agents used in this study for evaluation as an efflux pump inhibitor (EPI) on the activity of EtBr, MIC of each agent before and after 30th generation passage with sublethal concentration against *B. pseudomallei* strain 1026b, H777 and 316c. Data represent three independent experiments.

Bp Strain	Agents Used for Evaluation as an EPI	MIC Before Passage				MIC After 30th Passage			
		MIC of Agent (µg/mL)	MIC of EtBr (µg/mL)		Reduction (*n*-fold) in EtBr MIC	MIC of Agent (µg/mL)	MIC of EtBr (µg/mL)		Reduction (*n*-fold) in EtBr MIC
			Without Agent	With Agent			Without Agent	With Agent	
1026b	Ceftazidime	2	128	32	4	2	128	32	4
	AgNPs	32	128	8	16	32	128	8	16
H777	Ceftazidime	4	128	32	4	4	128	32	4
	AgNPs	64	128	16	8	64	128	16	8
316c	Ceftazidime	64	128	32	4	64	128	32	4
	AgNPs	64	128	32	4	64	128	32	4

## Data Availability

The data presented in this study are available on the request from the corresponding author.

## Supplementary Materials

The following are available online. Figure S1: Culture purity on Ashdown’s media and colony morphology of *B. pseudomallei*: (a) strain 1026b in 10th; (b) 20th; (c) 30th passage. Strain H777: (d) 10th; (e) 20th; (f) 30th passage. Strain 316c: (g) 10th; (h) 20th; (i) 30th passage.

## Sample Availability

Samples of the antibacterial agents assayed are available from the authors.

## References

[1] Hesstvedt L., Reikvam D.H., Dunlop O. (2015). Neurological melioidosis in Norway presenting with a cerebral abscess. IDCases.

[2] Wongkaewkhiaw S., Taweechaisupapong S., Anutrakunchai C., Nazmi K., Bolscher J.G., Wongratanacheewin S., Kanthawong S. (2019). D-LL-31 in combination with ceftazidime synergistically enhances bactericidal activity and biofilm destruction inBurkholderia pseudomallei. Biofouling.

[3] Kanthawong S., Bolscher J.G., Veerman E.C., Van Marle J., De Soet H.J., Nazmi K., Wongratanacheewin S., Taweechaisupapong S. (2012). Antimicrobial and antibiofilm activity of LL-37 and its truncated variants against Burkholderia pseudomallei. Int. J. Antimicrob. Agents.

[4] Fjell C.D., Hiss J.A., Hancock R.E.W., Schneider G. (2011). Designing antimicrobial peptides: Form follows function. Nat. Rev. Drug Discov..

[5] Inglis T.J.J., Rodrigues F., Rigby P., Norton R., Currie B.J. (2004). Comparison of the Susceptibilities of Burkholderia pseudomallei to Meropenem and Ceftazidime by Conventional and Intracellular Methods. Antimicrob. Agents Chemother..

[6] Sarovich D.S., Price E.P., Peacock S.J., Cook J.M., Schulze V., Wolken S.R., Keim P., Pearson T., Limmathurotsakul D. (2012). Development of ceftazidime resistance in an acute Burkholderia pseudomallei infection. Infect. Drug Resist..

[7] Ravindran A., Chandran P., Khan S.S. (2013). Biofunctionalized silver nanoparticles: Advances and prospects. Colloids Surf. B Biointerfaces.

[8] Soliman H., Elsayed A., Dyaa A. (2018). Antimicrobial activity of silver nanoparticles biosynthesised by Rhodotorula sp. strain ATL72. Egypt. J. Basic Appl. Sci..

[9] Graf C., Vossen D.L.J., Imhof A., Van Blaaderen A. (2003). A General Method To Coat Colloidal Particles with Silica. Langmuir.

[10] Chen X., Schluesener H.J. (2008). Nanosilver: A nanoproduct in medical application. Toxicol. Lett..

[11] Pal S., Tak Y.K., Song J.M. (2007). Does the Antibacterial Activity of Silver Nanoparticles Depend on the Shape of the Nanoparticle? A Study of the Gram-Negative Bacterium Escherichia coli. Appl. Environ. Microbiol..

[12] Van Dong P., Ha C.H., Binh L.T., Kasbohm J. (2012). Chemical synthesis and antibacterial activity of novel-shaped silver nanoparticles. Int. Nano Lett..

[13] Siritongsuk P., Hongsing N., Thammawithan S., Daduang S., Klaynongsruang S., Tuanyok A., Patramanon R. (2016). Two-Phase Bactericidal Mechanism of Silver Nanoparticles against Burkholderia pseudomallei. PLoS ONE.

[14] Markowska K., Grudniak A.M., I Wolska K. (2013). Silver nanoparticles as an alternative strategy against bacterial biofilms. Acta Biochim. Pol..

[15] Fabrega J., Renshaw J.C., Lead J.R. (2009). Interactions of Silver Nanoparticles withPseudomonas putidaBiofilms. Environ. Sci. Technol..

[16] Kalishwaralal K., BarathManiKanth S., Pandian S.R.K., Deepak V., Gurunathan S. (2010). Silver nanoparticles impede the biofilm formation by Pseudomonas aeruginosa and Staphylococcus epidermidis. Colloids Surf. B Biointerfaces.

[17] Martinez-Gutierrez F., Boegli L., Agostinho A., Sánchez E.M., Bach H., Ruiz F., James G. (2013). Anti-biofilm activity of silver nanoparticles against different microorganisms. Biofouling.

[18] Zhang X.-F., Liu Z.-G., Shen W., Gurunathan S. (2016). Silver Nanoparticles: Synthesis, Characterization, Properties, Applications, and Therapeutic Approaches. Int. J. Mol. Sci..

[19] Kim T.Y., Cha S.-H., Cho S., Park Y. (2016). Tannic acid-mediated green synthesis of antibacterial silver nanoparticles. Arch. Pharmacal Res..

[20] Myint K.B., Sing L.C., Wei Z. (2013). Tannic Acid as Phytochemical Potentiator for Antibiotic Resistance Adaptation. APCBEE Procedia.

[21] Sharma G., Nam J.-S., Sharma A.R., Lee S.-S. (2018). Antimicrobial Potential of Silver Nanoparticles Synthesized Using Medicinal Herb Coptidis rhizome. Molecules.

[22] Moteriya P., Padalia H., Chanda S. (2017). Characterization, synergistic antibacterial and free radical scavenging efficacy of silver nanoparticles synthesized using Cassia roxburghii leaf extract. J. Genet. Eng. Biotechnol..

[23] Danaei M., Dehghankhold M., Ataei S., Davarani F.H., Javanmard R., Dokhani A., Khorasani S., Mozafari M.R. (2018). Impact of Particle Size and Polydispersity Index on the Clinical Applications of Lipidic Nanocarrier Systems. Pharmaceutics.

[24] Wypij M., Jędrzejewski T., Ostrowski M., Trzcińska J., Rai M., Golińska P. (2020). Biogenic Silver Nanoparticles: Assessment of Their Cytotoxicity, Genotoxicity and Study of Capping Proteins. Molecules.

[25] Maillard A.P.F., Gonçalves S., Santos N.C., De Mishima B.A.L., Dalmasso P.R., Hollmann A. (2019). Studies on interaction of green silver nanoparticles with whole bacteria by surface characterization techniques. Biochim. Biophys. Acta Biomembr..

[26] Arya S.S., Sharma M.M., Das R.K., Rookes J., Cahill D., Lenka S.K. (2019). Vanillin mediated green synthesis and application of gold nanoparticles for reversal of antimicrobial resistance in Pseudomonas aeruginosa clinical isolates. Heliyon.

[27] Christena L.R., Mangalagowri V., Pradheeba P., Ahmed K.B.A., Shalini B.I.S., Vidyalakshmi M., Anbazhagan V., Subramanian N.S. (2015). Copper nanoparticles as an efflux pump inhibitor to tackle drug resistant bacteria. RSC Adv..

[28] Behdad R., Pargol M., Mirzaie A., Karizi S.Z., Noorbazargan H., Akbarzadeh I. (2020). Efflux pump inhibitory activity of biologically synthesized silver nanoparticles against multidrug-resistant Acinetobacter baumannii clinical isolates. J. Basic Microbiol..

[29] Fatima F., Verma S.R., Pathak N., Bajpai P. (2016). Extracellular mycosynthesis of silver nanoparticles and their microbicidal activity. J. Glob. Antimicrob. Resist..

[30] Rai M., Deshmukh S., Ingle A., Gade A. (2012). Silver nanoparticles: The powerful nanoweapon against multidrug-resistant bacteria. J. Appl. Microbiol..

[31] Cao Y., Zheng R., Ji X., Liu H., Xie R., Yang W. (2014). Syntheses and Characterization of Nearly Monodispersed, Size-Tunable Silver Nanoparticles over a Wide Size Range of 7–200 nm by Tannic Acid Reduction. Langmuir.

[32] Cataldo F., Angelini G. (2013). A Green Synthesis of Colloidal Silver Nanoparticles and Their Reaction with Ozone. Eur. Chem. Bull..

[33] Ahmad T. (2014). Reviewing the Tannic Acid Mediated Synthesis of Metal Nanoparticles. J. Nanotechnol..

[34] Chaturvedi V.K., Yadav N., Rai N.K., Ellah N.H.A., Bohara R.A., Rehan I.F., Marraiki N., Batiha G.E.-S., Hetta H.F., Singh P. (2020). *Pleurotus sajor-caju*-Mediated Synthesis of Silver and Gold Nanoparticles Active against Colon Cancer Cell Lines: A New Era of Herbonanoceutics. Molecules.

[35] Abudalo M.A., Al-Mheidat I.R., Al-Shurafat A.W., Grinham C., Oyanedel-Craver V. (2019). Synthesis of silver nanoparticles using a modified Tollens’ method in conjunction with phytochemicals and assessment of their antimicrobial activity. PeerJ.

[36] Ajitha B., Reddy Y.A.K., Reddy P.S., Jeon H.-J., Ahn C.W. (2016). Role of capping agents in controlling silver nanoparticles size, antibacterial activity and potential application as optical hydrogen peroxide sensor. RSC Adv..

[37] Orlowski P., Tomaszewska E., Ranoszek-Soliwoda K., Gniadek M., Labedz O., Malewski T., Nowakowska J., Chodaczek G., Celichowski G., Grobelny J. (2018). Tannic Acid-Modified Silver and Gold Nanoparticles as Novel Stimulators of Dendritic Cells Activation. Front. Immunol..

[38] Morones J.R., Elechiguerra J.L., Camacho A., Holt K., Kouri J.B., Ramírez J.T., Yacaman M.J. (2005). The bactericidal effect of silver nanoparticles. Nanotechnology.

[39] Liu L., Ge C., Zhang Y., Ma W., Su X., Chen L., Li S., Wang L., Mu X., Xu Y. (2020). Tannic acid-modified silver nanoparticles for enhancing anti-biofilm activities and modulating biofilm formation. Biomater. Sci..

[40] Lu Z., Rong K., Li J., Yang H., Chen R. (2013). Size-dependent antibacterial activities of silver nanoparticles against oral anaerobic pathogenic bacteria. J. Mater. Sci. Mater. Med..

[41] Raza M.A., Kanwal Z., Rauf A., Sabri A.N., Riaz S., Naseem S. (2016). Size- and Shape-Dependent Antibacterial Studies of Silver Nanoparticles Synthesized by Wet Chemical Routes. Nanomaterials.

[42] A Skomorokhova E., Sankova T.P., A Orlov I., Savelev A.N., Magazenkova D.N., Pliss M.G., Skvortsov A.N., Sosnin I.M., A Kirilenko D., Grishchuk I.V. (2020). Size-Dependent Bioactivity of Silver Nanoparticles: Antibacterial Properties, Influence on Copper Status in Mice, and Whole-Body Turnover. Nanotechnol. Sci. Appl..

[43] Feng Q.L., Chen G.Q., Cui F.Z., Kim T.N., Kim J.O. (2000). A Mechanistic Study of the Antibacterial Effect of Silver Ions on Escherichia Coli and Staphylococcus Aureus. J. Biomed. Mater. Res..

[44] Ipe D.S., Kumar P.T.S., Love R.M., Hamlet S.M. (2020). Silver Nanoparticles at Biocompatible Dosage Synergistically Increases Bacterial Susceptibility to Antibiotics. Front. Microbiol..

[45] Bakhtiari-Sardari A., Mashreghi M., Eshghi H., Behnam-Rasouli F., Lashani E., Shahnavaz B. (2020). Comparative evaluation of silver nanoparticles biosynthesis by two cold-tolerant Streptomyces strains and their biological activities. Biotechnol. Lett..

[46] Shahmiri M., Enciso M., Adda C.G., Smith B.J., Perugini M.A., Mechler A. (2016). Membrane Core-Specific Antimicrobial Action of Cathelicidin LL-37 Peptide Switches Between Pore and Nanofibre Formation. Sci. Rep..

[47] Kuroda K., Okumura K., Isogai H., Isogai E. (2015). The Human Cathelicidin Antimicrobial Peptide LL-37 and Mimics are Potential Anticancer Drugs. Front. Oncol..

[48] Anantharaman A., Rizvi M.S., Sahal D. (2010). Synergy with Rifampin and Kanamycin Enhances Potency, Kill Kinetics, and Selectivity of De Novo-Designed Antimicrobial Peptides. Antimicrob. Agents Chemother..

[49] Foerster S., Unemo M., Hathaway L.J., Low N., Althaus C.L. (2016). Time-kill curve analysis and pharmacodynamic modelling for in vitro evaluation of antimicrobials against Neisseria gonorrhoeae. BMC Microbiol..

[50] Vorachit M., Lam K., Jayanetra P., Costerton J.W. (1995). Electron Microscopy of the Mode of Growth of Pseudomonas Psedomallei in Vitro and in Vivo. J. Trop. Med. Hyg..

[51] Sawasdidoln C., Taweechaisupapong S., Sermswan R.W., Tattawasart U., Tungpradabkul S., Wongratanacheewin S. (2010). Growing Burkholderia pseudomallei in Biofilm Stimulating Conditions Significantly Induces Antimicrobial Resistance. PLoS ONE.

[52] Palanisamy N.K., Ferina N., Amirulhusni A.N., Mohd-Zain Z., Hussaini J., Ping L.J., Durairaj R. (2014). Antibiofilm properties of chemically synthesized silver nanoparticles found against Pseudomonas aeruginosa. J. Nanobiotechnol..

[53] Jiang X., Yu T., Xu P., Xu X., Ji S., Gao W., Shi L. (2018). Role of Efflux Pumps in the in vitro Development of Ciprofloxacin Resistance in Listeria monocytogenes. Front. Microbiol..

[54] Anbazhagan P.V., Thavitiki P.R., Varra M., Annamalai L., Putturu R., Lakkineni V.R., Pesingi P.K. (2019). Evaluation of efflux pump activity of multidrug-resistant Salmonella Typhimurium isolated from poultry wet markets in India. Infect. Drug Resist..

[55] Podnecky N.L., Rhodes K.A., Schweizer H.P. (2015). Efflux pump-mediated drug resistance in Burkholderia. Front. Microbiol..

[56] Pathania R., Sharma A., Gupta V.K. (2019). Efflux pump inhibitors for bacterial pathogens: From bench to bedside. Indian J. Med Res..

[57] Chatterjee A.K., Chakraborty R., Basu T. (2014). Mechanism of antibacterial activity of copper nanoparticles. Nanotechnology.

[58] Mishra M., Kumar S., Majhi R.K., Goswami L., Goswami C., Mohapatra H. (2018). Antibacterial Efficacy of Polysaccharide Capped Silver Nanoparticles Is Not Compromised by AcrAB-TolC Efflux Pump. Front. Microbiol..

[59] Nallathamby P.D., Lee K.J., Desai T., Xu X.-H.N. (2010). Study of the Multidrug Membrane Transporter of Single LivingPseudomonas aeruginosaCells Using Size-Dependent Plasmonic Nanoparticle Optical Probes. Biochemistry.

[60] Salas-Orozco M., Niño-Martínez N., Martínez-Castañón G.-A., Méndez F.T., Jasso M.E.C., Ruiz F. (2019). Mechanisms of Resistance to Silver Nanoparticles in Endodontic Bacteria: A Literature Review. J. Nanomater..

[61] Dakal T.C., Kumar A., Majumdar R.S., Yadav V. (2016). Mechanistic Basis of Antimicrobial Actions of Silver Nanoparticles. Front. Microbiol..

[62] Dobias J., Bernier-Latmani R. (2013). Silver Release from Silver Nanoparticles in Natural Waters. Environ. Sci. Technol..

[63] Clinical and Laboratory Standards Institute (CLSI) (2013). Performance Standards for Antimicrobial Susceptibility Testing; Twenty-Third Informational Supplement.

[64] Irazazabal L.N., Porto W.F., Fensterseifer I.C., Alves E.S., Matos C.O., Menezes A.C., Felício M.R., Gonçalves S., Santos N.C., Ribeiro S.M. (2019). Fast and potent bactericidal membrane lytic activity of PaDBS1R1, a novel cationic antimicrobial peptide. Biochim. Biophys. Acta Biomembr..

[65] Silveira Z.D.S., Macêdo N.S., Dos Santos J.F.S., De Freitas T.S., Barbosa C.R.D.S., Júnior D.L.D.S., Muniz D.F., De Oliveira L.C.C., Júnior J.P.S., Da Cunha F.A.B. (2020). Evaluation of the Antibacterial Activity and Efflux Pump Reversal of Thymol and Carvacrol against Staphylococcus aureus and Their Toxicity in Drosophila melanogaster. Molecules.

[66] Teh C.H., Nazni W.A., Nurulhusna A.H., Norazah A., Lee H.L. (2017). Determination of antibacterial activity and minimum inhibitory concentration of larval extract of fly via resazurin-based turbidometric assay. BMC Microbiol..

[67] Sengyee S., Saiprom N., Paksanont S., Limmathurotsakul D., Wuthiekanun V., Chantratita N. (2017). Susceptibility of Clinical Isolates of Burkholderia pseudomallei to a Lipid A Biosynthesis Inhibitor. Am. J. Trop. Med. Hyg..

[68] Pankey G.A., Ashcraft D.S. (2009). The detection of synergy between meropenem and polymyxin B against meropenem-resistant Acinetobacter baumannii using Etest® and time-kill assay. Diagn. Microbiol. Infect. Dis..

[69] Kunyanee C., Kamjumphol W., Taweechaisupapong S., Kanthawong S., Wongwajana S., Wongratanacheewin S., Hahnvajanawong C., Chareonsudjai S. (2016). Burkholderia pseudomallei Biofilm Promotes Adhesion, Internalization and Stimulates Proinflammatory Cytokines in Human Epithelial A549 Cells. PLoS ONE.

[70] Lemos A.S.O., Campos L.M., Melo L., Guedes M.C.M.R., Oliveira L.G., Silva T.P., Melo R.C.N., Rocha V.N., Aguiar J.A.K., Apolônio A.C.M. (2018). Antibacterial and Antibiofilm Activities of Psychorubrin, a Pyranonaphthoquinone Isolated From Mitracarpus frigidus (Rubiaceae). Front. Microbiol..

[71] Elbehiry A., Al-Dubaib M., Marzouk E., Moussa I. (2019). Antibacterial effects and resistance induction of silver and gold nanoparticles againstStaphylococcus aureus-induced mastitis and the potential toxicity in rats. Microbiol. Open.

