# Supplementary figures and images for: Tannic Acid-Stabilized Silver Nanoparticles Used in Biomedical Application as an Effective Antimelioidosis and Prolonged Efflux Pump Inhibitor against Melioidosis Causative Pathogen
Source: Molecules. 2021 Feb 14;26(4):1004. doi: 10.3390/molecules26041004 (PMC7918740; doi:10.3390/molecules26041004)

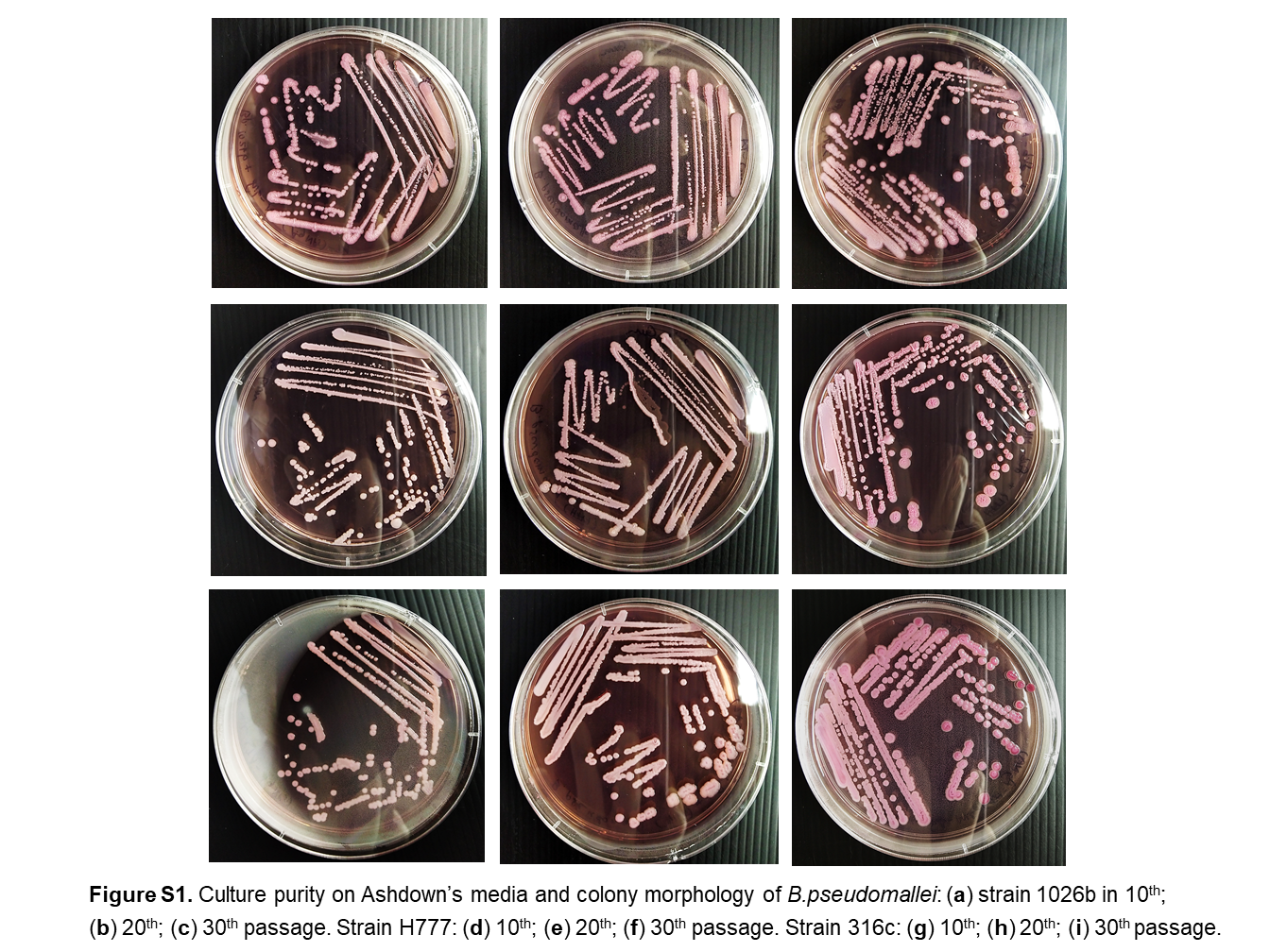

Supplement: Supplementary file 1 [file molecules-26-01004-s001.zip › Supplementary materials/Figure S1.tif]
